# Usefulness of NT-pro BNP monitoring to identify echocardiographic responders following cardiac resynchronization therapy

**DOI:** 10.1186/1476-7120-7-39

**Published:** 2009-08-20

**Authors:** Julien Magne, Michelle Dubois, Jean Champagne, Jean G Dumesnil, Philippe Pibarot, François Philippon, Gilles O'Hara, Mario Sénéchal

**Affiliations:** 1 Institut Universitaire de Cardiologie et de Pneumologie de Québec, Department of Cardiology, Quebec, Canada

## Abstract

**Background:**

Cardiac resynchronization therapy (CRT) improves left ventricular (LV) volumes, mitral regurgitation (MR) severity and symptoms of patients with heart failure (HF). However, ≥ 30% of patients have no significant clinical or echocardiographic improvement following CRT. Reverse remodeling after CRT correlates with improved clinical outcomes. We hypothesized that in NT-pro BNP monitoring is accurate to identify responders following CRT.

**Methods:**

42 consecutive patients (mean age 66 ± 12 years, male 68%) with HF undergoing CRT were prospectively enrolled. Responders at follow-up were defined by echocardiography (decrease in LV end systolic volume ≥ 15%). Echocardiography and NT-pro BNP measurement were performed at baseline and repeated 3 to 6 month after CRT.

**Results:**

There was no significant difference between responders (n = 29, 69%) and non-responders (n = 13, 31%) regarding baseline NT-pro BNP level. Responders had significantly higher decrease in NT-pro BNP levels during follow-up than non-responders (absolute: -1428 ± 1333 pg.ml^-1 ^vs. -61 ± 959 pg.ml^-1^, p = 0.002; relative: -45 ± 28% vs. 2 ± 28%, p < 0.0001). A decrease of ≥ 15% in NT-pro BNP 3–6 months after CRT identifies echocardiographic responders with a sensitivity of 90% and a specificity of 77%.

**Conclusion:**

NT-pro BNP monitoring can accurately identify echocardiographic responders after CRT.

## Background

Cardiac resynchronization therapy (CRT) is established as a valuable additive treatment for patients with drug-refractory heart failure (HF) and electromechanical asynchrony. CRT improves left ventricular (LV) function and geometry, exercise capacity and outcomes of appropriately selected patients with HF [1-4]. CRT also leads to a decrease in both resting and exercise mitral regurgitation severity [5,6] by enhancement of LV function and local synchronicity (i.e. mechanical activation of papillary muscle insertion sites) [7]. Remodeling is a predictor of cardiovascular mortality in patients with congestive HF [1,2]. Reverse remodeling through pharmacological intervention and CRT correlates with improved clinical outcomes [6]. An important issue in CRT is the problem of non-responders. Using clinical criteria, rate of non response to CRT are estimated to be 20–30%, but in studies using objective parameters of LV remodeling, CRT non-responder rate reaches 40–50% [8]. Brain natriuretic peptides (BNP) and its inactive aminoterminal portion (NT-pro BNP), are neurohormones released by the ventricle in response to increase LV wall stress. Hence, BNP level may have valuable role for the assessment of cardiac dysfunction, particularly LV dysfunction, and for the monitoring of the response to cardiac therapy [9,10]. Recently, Fruhwald et al[11] showed that CRT leads to an early and sustained decrease in NT-pro BNP potentially reflecting improvement in LV function. In responders, left lateral wall pacing increases systolic function, reduces mitral regurgitation (MR) and thus decrease the wall motion stress. In this favorable remodeling process, neurohumoral activity is reduced and the decrease in plasma B-type natriuretic peptide after initiation of CRT predicts clinical improvement during follow-up [12]. We hypothesized that NT-pro BNP reduction following CRT correlates with LV remodeling and MR improvement and that change in NT-pro BNP following CRT can identify echocardiographic responders with good accuracy.

## Methods

From May 2005 to March 2008, 42 consecutive patients referred for CRT with chronic HF were prospectively enrolled. Inclusion criteria were as follow: (1) NYHA functional class ≥ III, (2) QRS duration ≥ 120 ms, (3) chronic LV systolic dysfunction defined as LV ejection fraction (LVEF) ≤ 35%, (4) LV dyssynchrony ≥ 50 ms, (5) optimal medical treatment for HF including angiotensin-converting enzyme inhibitors or AT1 receptor antagonists diuretics, beta-receptor blockers and spironolactone when tolerated, and (6) sinus rhythm. Patients with recent myocardial infarction (< 6 months), coronary revascularization procedure (< 6 months) and presenting standard contraindications to DSE were excluded. This study complies with the "*Declaration of Helsinki" *and all patients provided informed consent. The study protocol was approved by local ethics committee.

### Protocol

Patients were submitted to clinical examination, 12-lead electrocardiography (EKG), blood sampling, transthoracic echocardiography (TTE), and dobutamine stress echocardiography (DSE) within one week before CRT. Additionally, TTE and blood sampling were also performed within the week and at 3 to 6 month following CRT.

### Doppler echocardiography and DSE

Two-dimensional and Doppler TTE examination were performed with commercially available echocardiographic systems (Sonos 5500 or 7500, Philips Medical Systems, Amsterdam, The Netherlands). Doppler-echocardiographic measurements included LV end-diastolic and end-systolic diameters and LVEF determined by modified biplane Simpson method. LV outflow tract stroke volume was calculated by multiplying LV outflow tract area by LV outflow tract velocity-time integral measured by pulsed wave Doppler. Proximal isovelocity surface area (PISA) method was used to quantify both mitral regurgitation volume (RV) and effective regurgitant orifice (ERO) area as recommended by The American Society of Echocardiography guideline [13]. Quantification of interventricular asynchronism was obtained by recording aortic and pulmonary Doppler flows with pulsed wave, from the apical four-chamber and parasternal short-axis views respectively. Aortic and pulmonary ejection delays were defined as the delay between the onset of the QRS complex on the surface EKG and the onset of the aortic and pulmonary waves. Intraventricular asynchronism measurement was assessed with tissue Doppler imaging (TDI) from apical views to evaluate longitudinal myocardial regional function, analyzing septal, inferior, lateral, anterior and posterior walls. Velocity profiles were recorded with a sample volume placed in the middle of the basal segment of each LV wall. TDI signals were recorded at a sweep of 100 mm/s. Intraventricular asynchronism was defined as the time difference between the shortest and longest electromechanical delay among the five LV walls [14]. Responders were defined as a post-CRT decrease in LV end-systolic volume ≥ 15% at the 3–6 months follow-up echocardiography [14]. The wall motion score index (WMSI) was quantified at rest and during DSE, using a 16-segment model as recommended [13]. DSE was performed according to a low-dose infusion protocol. Patients received 5, 10, 15, and 20 μg/kg/min of dobutamine in 3-minutes stages, with echocardiographic images recorded at the end of each stage. The presence of LV contractile reserve (CR) was defined as an improvement of ≥ 0.20 in WMSI (rest-DSE). Heart rate and blood pressure were monitored during each stage. Criteria for stopping dobutamine infusion were (1) hypotension (systolic blood pressure < 90 mmHg), (2) angina, (3) significant arrhythmias (atrial fibrillation, bigeminy, ventricular tachycardia), (4) achievement of 85% maximal predicted heart rate.

### CRT implantation

A coronary sinus venogram was obtained using balloon catheter, followed by the insertion of the LV pacing lead. An SF guiding catheter was used to position the LV lead (Guidant Corporation, St Paul, NM or Medtronic Inc, Minneapolis, MN) in the coronary sinus.

The preferred position was lateral or postero-lateral vein. Right atrial and ventricular leads were positioned conventionally. All leads were connected to a dual-chamber biventricular ICD (Guidant Corporation, or Medtronic Inc).

### Blood sampling and NT-pro BNP measurement

Venous blood samples were withdrawn from an antecubital vein into chilled ethylene-diamine-tetra-acetic acid Vacutainer test tubes after 20 minutes of rest with patients in a supine position. Samples were placed immediately on ice-cold water and the tubes were then centrifuged at 4000 r.p.m. at 4°C for 15 min. Supernatant plasma was then immediately aliquoted into labelled cryo-vials. NT-pro BNP was determined by a commercially available electrochemiluminescence immunoassay based on a polyclonal antibody-based sandwich chemiluminescence assay (Roche Diagnostics, Germany) using an autoanalyser (Elecsys 2010).

### Statistical analysis

Continuous variables are expressed as mean ± SD or mean ± SEM when specified. Patients were separated into 2 groups (responders and non-responders). Baseline data for responders versus non-responders groups were compared for statistical significance using t-test or chi-square test, as appropriate. Baseline and post-CRT Doppler-echocardiographic data were analyzed using a 2-way analysis of variance for repeated measurements to assess the effects of time (baseline vs. post-CRT) and group (non-responders vs. responders). Linear regression analyses were used to evaluate the relationship between changes in NT-pro BNP and changes in echocardiographic parameters. Sensitivity and specificity for identification of CRT responders were determined for various cut-off values of echocardiographic parameters using receiver-operating characteristic (ROC) curves.

Forward and backward multiple stepwise regression were performed to determine the association between the change in NT-pro BNP and LV end-systolic volume after adjustment for baseline variables. Logistic regression was also performed to assess the performance of change in NT-pro BNP to identify CRT responders.

## Results

Among the 42 patients included in this study (mean age 66 ± 12 years, male 68%), 29 (69%) were responders. There was no significant difference between responders and non-responders (Table 1) regarding to age, prevalence of male gender, coronary artery disease, functional NYHA class III or IV and left or right bundle branch block at baseline. Although non-responders had significantly more frequent intraventricular conduction defect than responders (p = 0.04), there was no significant difference between the 2 groups regarding pre-CRT pacing and PR duration (Table 1). No significant difference between groups was found for baseline and post CRT medication at last follow-up.

**Table 1:** Baseline demographic and clinical data.

**Variables**	**All patients (n = 42)**	**Responders (n = 29, 69%)**	**Non-Responders (n = 13, 31%)**	**P Value**
**Demographic data**				
Age, years	66 ± 12	66 ± 11	66 ± 15	0.83
Male, n (%)	30 (68)	19 (65)	10 (77)	0.45
CAD, n (%)	30 (68)	18 (62)	11 (85)	0.13
**Clinical data**				
QRS duration, ms	159 ± 28	162 ± 27	155 ± 30	0.51
LBBB, n (%)	27 (61)	19 (65)	6 (46)	0.24
RBBB, n (%)	2 (4.5)	2 (7)	0 (0)	...
IVCD, n (%)	8 (18)	3 (10)	5 (38)	0.04
PR, ms	180 ± 40	172 ± 32	195 ± 51	0.08
Pre-CRT pacing, n (%)	7 (16)	5 (17)	2 (15)	0.88
NYHA III/IV, n (%)	31 (70)/13 (30)	22 (76)/7 (24)	7 (54)/6 (46)	0.16
**Medication**				
Diuretic, n (%)	41 (93)	27 (93)	12 (92)	0.93
β-Blockers, n (%)	41 (93)	26 (90)	13 (100)	0.13
ACEi, n (%)	31 (70)	20 (69)	10 (77)	0.59
AR Blockers, n (%)	13 (30)	9 (32)	3 (23)	0.55
Digoxin, n (%)	11 (25)	5 (17)	6 (46)	0.06
Spironolactone, n (%)	26 (59)	15 (52)	10 (77)	0.12

CAD indicates coronary arteries disease, LBBB, left bundle branch block, RBBB, right bundle branch block, IVCD, intraventricular conduction defect, ACEi, angiotensin converting enzyme inhibitors and AR, angiotensin receptors.

### NT-pro BNP at baseline and follow-up

**Figure 1:**
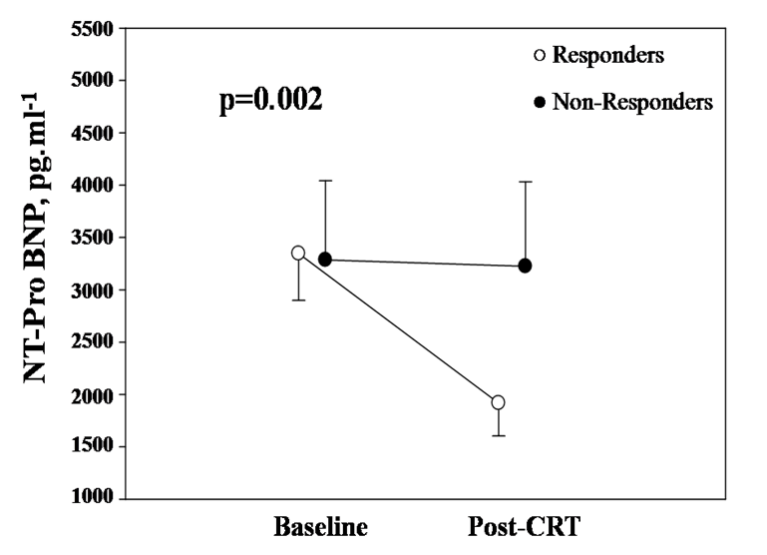
**Changes in Nt-pro BNP in responders and non responders defined as a decrease ≥ 15% in LV end systolic volume 3 to 6 months post CRT**. Data are expressed as mean ± SEM.

The decrease in NT-pro BNP following CRT was higher in responders than in non-responders (p = 0.002) (Figure 1). Baseline mean NT-pro BNP was 3328 ± 2474 pg.ml^-1 ^(25% quartile = 1186 pg.ml^-1^, median = 2776 pg.ml^-1^, 75% quartile = 5098 pg.ml^-1^) and was not statistically different between responders and non-responders (Figure 1) (3346 ± 2401 pg.ml^-1 ^and 3286 ± 695 pg.ml^-1^, p = 0.95). The change in NT-pro BNP between pre-CRT and 3–6 months post CRT evaluations was significantly more important in responders than in non-responders (absolute: -1428 ± 1333pg.ml^-1 ^vs.-61 ± 959 pg.ml^-1^, p = 0.002; relative: -45 ± 28% vs. 2 ± 28%, p < 0.0001). Moreover, there was a definite trend for significant difference between the 2 groups with regards to 3–6 months NT-pro BNP (p = 0.07).

### Baseline and chronic changes in echocardiographic parameters

Baseline LV volumes, LVEF, resting LV WMSI, MR severity (i.e. ERO, RV and prevalence of severe MR) and inter and intraventricular asynchronism were not significantly different between responders and non-responders (Table 2). However, non-responder patients had higher baseline LV stroke volume than responders but after CRT this difference was no longer significant. As expected, LV geometry and function as well as MR severity were significantly improved in responders following CRT (Table 2). By definition, patients with response to CRT had better improvement of LV end-systolic volume compared to non-responders (-42 ± 17 ml vs. 13 ± 24 ml, p < 0.0001 and -26 ± 10% vs. -6 ± 10%, p < 0.0001).

**Table 2:** Baseline and late post-CRT Echocardiographic data.

**Variables**	**All patients (n = 42)**	**Responders (n = 29, 69%)**	**Non Responders (n = 13, 31%)**	**P Value**
**LV geometry and function**				
LV end-systolic Volume, ml				
Baseline	177 ± 65	175 ± 64	189 ± 62	0.49
Late post-CRT	155 ± 67	133 ± 57*	198 ± 67	0.0017
LV end-diastolic Volume, ml				
Baseline	213 ± 69	209 ± 67	228 ± 67	0.40
Late post-CRT	201 ± 73	181 ± 66*	239 ± 72*	0.01
LV stroke Volume, ml				
Baseline	42 ± 13	39 ± 10	50 ± 13	0.0048
Late post-CRT	51 ± 11	51 ± 9*	49 ± 14	0.65
LV ejection fraction, %				
Baseline	19 ± 7	18 ± 7	19 ± 7	0.75
Late post-CRT	24 ± 9	27 ± 9*	18 ± 6	0.0025
Wall Motion Score Index				
Baseline rest	3.5 ± 0.4	3.4 ± 0.4	3.6 ± 0.2	0.29
Baseline dobutamine	3.1 ± 0.4	3 ± 0.4*	3.3 ± 0.4*	0.038
**Contractile Reserve, n (%)**	30 (71)	24 (83)	6 (46)	0.02
**Mitral Regurgitation**				
Effective orifice Area, mm^2^				
Baseline	18 ± 13	18 ± 12	17 ± 14	0.73
Late post-CRT	10 ± 11	5 ± 5*	19 ± 15	< 0.0001
Regurgitant Volume, ml				
Baseline	29 ± 26	31 ± 29	25 ± 19	0.54
Late post-CRT	17 ± 17	11 ± 13*	29 ± 19	0.0009
Severe Mitral Regurgitation, n (%)				
Baseline	19 (45)	12 (41)	7 (54)	0.45
Late post-CRT	10 (61)	2 (7)*	8 (61)	0.0005
**Asynchronism**				
Interventricular, ms	46 ± 27	46 ± 30	45 ± 22	0.92
Intraventricular, ms	85 ± 25	85 ± 25	85 ± 27	0.93

LV indicates left-ventricular and CRT, cardiac resynchronisation therapy. * significant difference (p < 0.05) between baseline and late post-CRT or between rest and dobutamine data; severe mitral regurgitation is defined as an effective orifice area ≥ 20 mm^2^.

### Contractile Reserve

CR was present in 30 patients (71%) and responders had significant higher prevalence of CR than non-responders (83% vs. 46%, p = 0.02) (Table 2). Consistently, the improvement in WMSI during DSE was more important in responders than in non-responders (13.2 ± 7.8% vs. -7.6 ± 9.2%, p = 0.048). Compared to patients without CR, those with CR had significant lower baseline and follow-up LV end-systolic volume (167 ± 56 vs. 213 ± 58 ml, p = 0.03 and 135 ± 52 vs. 207 ± 83 ml, p = 0.001, respectively). They also had higher decrease in LV end-systolic volume following CRT (-33 ± 5 vs. -6 ± 9 ml, p = 0.01).

According to the presence of CR, there was no significant difference in baseline and post-CRT NT-pro BNP (p = 0.69 and p = 0.26). Although there was no significant difference in baseline ERO (17 ± 9 vs. 22 ± 11 mm^2^, p = 0.22), patients with CR had lower ERO at follow-up (8 ± 7 vs. 20 ± 14 mm^2^, p = 0.004) than those without CR.

### Relations between changes in MR severity and echocardiographic parameters

**Figure 2:**
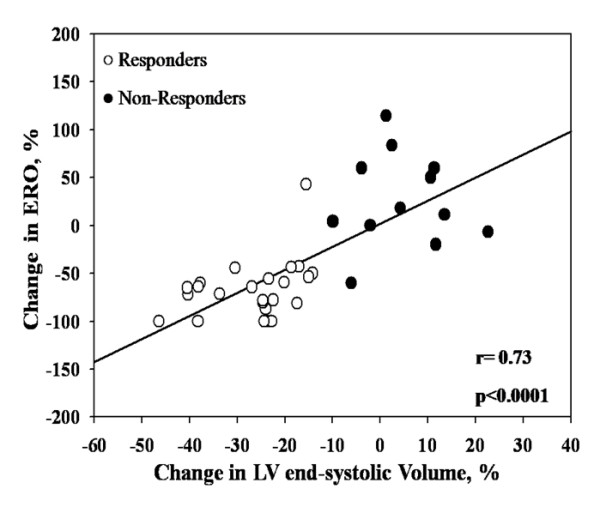
**Correlation between changes in effective regurgitant orifice (ERO) area and changes in LV end-systolic volumes**.

**Figure 3:**
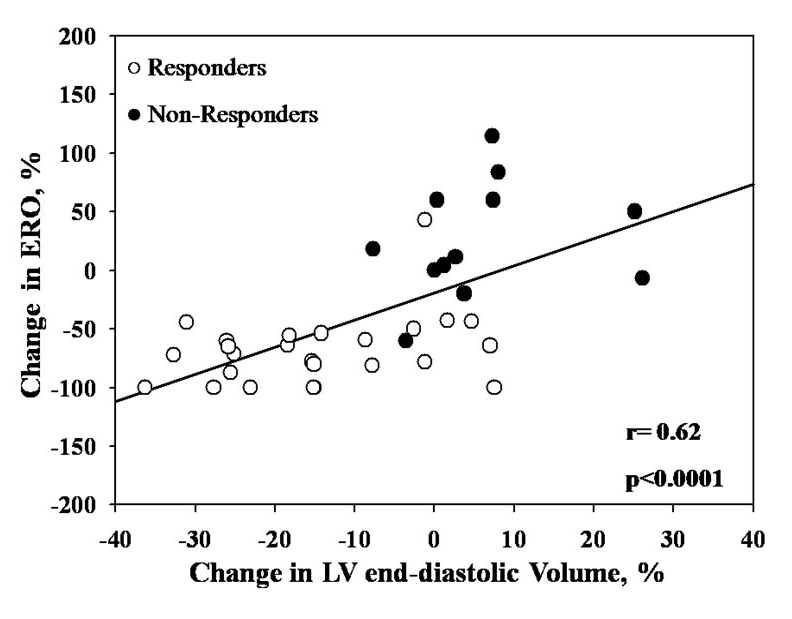
**Correlation between changes in effective regurgitant orifice (ERO) area and changes in LV end-diastolic volumes**.

Percent changes in ERO and in RV were correlated with percent changes in LV end-systolic volume (ERO: r = 0.73, p < 0.0001, RV: r = 0.53, p = 0.0008) (Figure 2) and end-diastolic volumes (ERO: r = 0.62, p < 0.0001, RV: r = 0.49, p = 0.002) (Figure 3).

However, only the change in ERO was correlated with the peak WMSI and the change in WMSI during DSE (r = 0.39, p = 0.018 and r = 0.42, p = 0.0098, respectively.

### Relations between changes in NT-pro BNP and echocardiographic parameters

**Figure 4:**
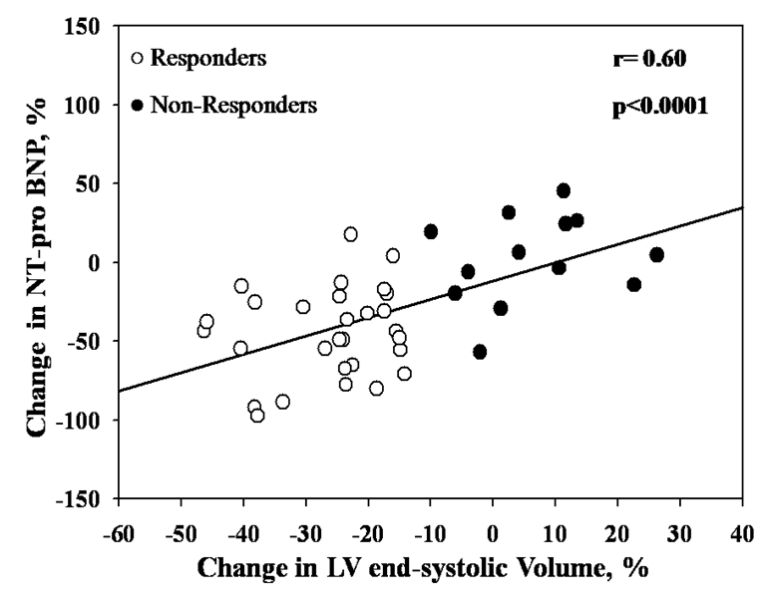
**Correlation between changes in NT-pro BNP and changes in LV end-systolic volume**.

**Figure 5:**
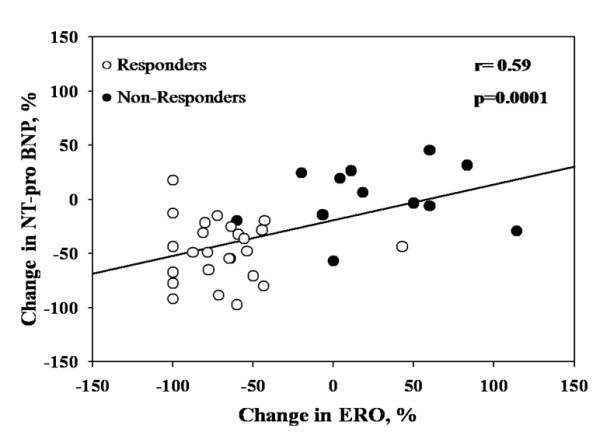
**Correlation between changes in NT-pro BNP and changes in effective regurgitant orifice (ERO) area**.

**Figure 6:**
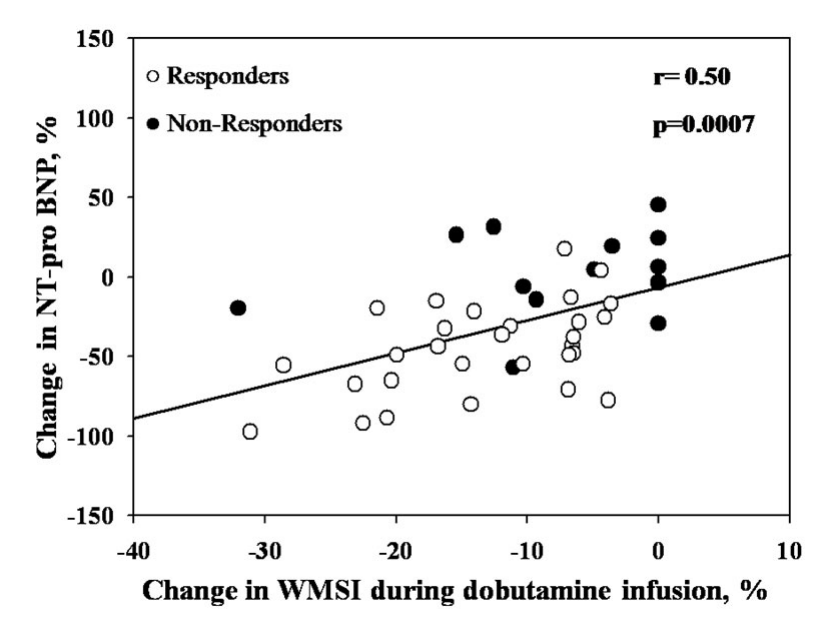
**Correlation between changes in NT-pro BNP and changes in LV wall motion score index (WMSI) during dobutamine stress echocardiography**.

The percent change in NT-pro BNP correlated with percent change in LVEF (r = 0.32, p = 0.038), LV stroke volume, (r = 0.31, p = 0.043), LV end-diastolic volume (r = 0.42, p = 0.006) and RV (r = 0.45, p = 0.003). The best correlations were found with percent changes in LV end-systolic volume (r = 0.60, p < 0.0001) and ERO (r = 0.59, p = 0.0001) (Figure 4 and 5). Moreover there was also a significant correlation between the percent change in WMSI during dobutamine infusion and percent change in NT-pro BNP (r = 0.50, p = 0.0007) (Figure 6). Patients with CR had higher changes and percent change in NT-pro BNP than those without CR (p = 0.0093 and p = 0.0047, respectively).

### Utilization of NT-pro BNP for identification of echocardiographic response to CRT and change in MR severity

**Figure 7:**
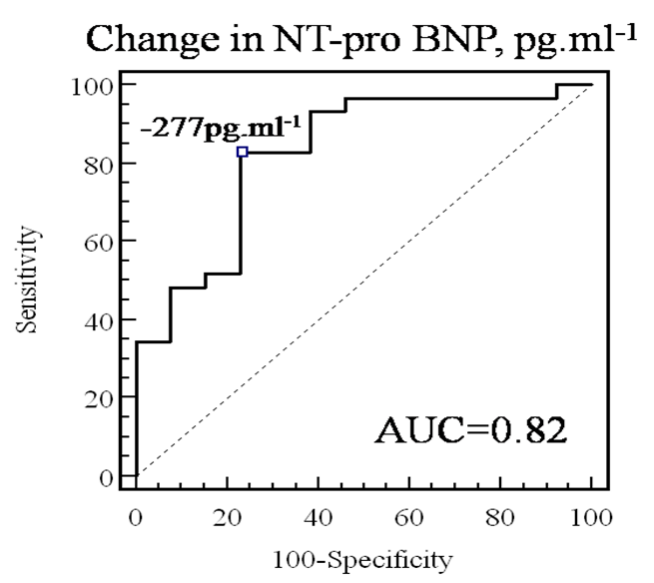
**Receiver-operating characteristic (ROC) curves analysing the accuracy of absolute changes in NT-pro BNP to identify CRT response**.

**Figure 8:**
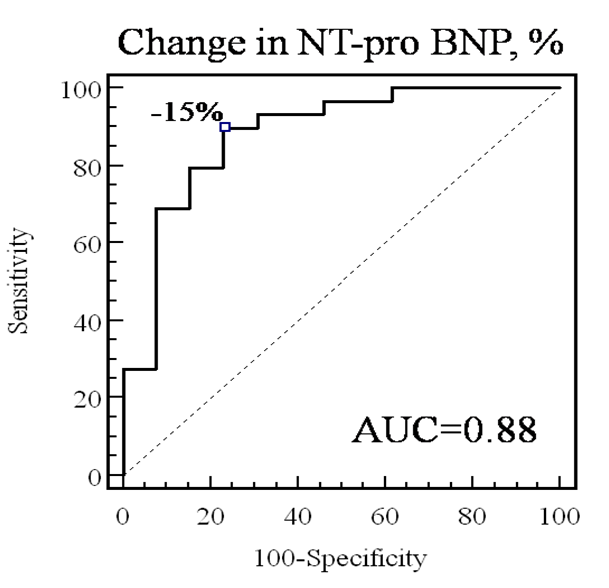
**Receiver-operating characteristic (ROC) curves analysing the accuracy of relative changes in NT-pro BNP to identify CRT response**.

After adjustment for age, change in NT-pro BNP remains correlated with the percent change in LV end-systolic volume (r = 0.58, p = 0.0001). ROC curve analysis, demonstrated that the absolute change in NT-pro BNP is accurate to identify response to CRT (area under curve = 0.82) and that a decrease in BNP ≥ 277 pg.ml^-1 ^identifies echocardiographic responders (Figure 7) with good sensitivity (83%), specificity (77%), positive predictive value (89%) and negative predictive value (64%). The percent change in NT-pro BNP was slightly more accurate (area under curve = 0.88) with a sensitivity of 90%, a specificity of 77%, a positive predictive value of 90% and a negative predictive value of 75%, for percent decrease NT-pro BNP ≥ 15% (Figure 8). In multivariate linear regression, after adjustment for age, sex, baseline LVEF and LV end-systolic volume, percent change in NT-pro BNP was independently associated with percent of change in LV end-systolic volume (β = 0.43 ± 0.14, p = 0.004).

Multivariate logistic regression showed, after adjustment for other baseline variables, that the percent change in NT-pro BNP is a good surrogate marker of response to CRT (odds-ratio = 1.08 (95% confidence interval: 1.03–1.16) per 1% of decrease in NT-pro BNP, p = 0.008). Similar results were found when adding CR in the multivariate models. When entering, the change in NT-pro BNP as a dichotomous variable in the multivariate logistic model, an absolute decrease in NT-pro BNP ≥ 277 pg.ml^-1 ^and a percent decrease of ≥ 15% were independently associated with a 9.4-fold (p = 0.0056) and 7.1-fold (p = 0.0018) increase in the likelihood of positive response to CRT, respectively. Multivariate linear regression also identified the percent change in NT-pro BNP ≥ 15%, after adjusting for baseline characteristics (i.e. age, sex, ERO and LV end-systolic volume), as strongly associated with the percent change in ERO (β = 37 ± 8.5, p < 0.0001).

## Discussion

The main finding of our study is that change in NT-pro BNP level between baseline and 3 to 6 months after CRT may be useful to identify (1) echocardiographic responders following CRT and (2) the magnitude of decrease in MR severity following CRT. Our results also confirm that change in MR severity after CRT implantation is related to the improvement in LV geometry. Furthermore, previous studies found that baseline LV viability is a strong predictor of LV performance following CRT [12-14]. Hence, correlation between the change in LV WMSI during DSE and the change in NT-pro BNP (Figure 4), emphasized by our data, suggests that a substantial amount of recruitable myocardium is needed to obtain improvement in LV function and MR and thus decrease in NT-pro BNP after CRT. Consistently, we also confirmed results from previous studies showing that the presence of CR is a good predictor [15-17] of response to CRT.

### Cardiac resynchronization therapy and brain natriuretic peptide

Several previous studies reported a significant decrease in natriuretic peptide following CRT implantation [8,18-24]. Recently, Fruhwald et al. [11] demonstrated, in patients with moderate or severe HF and LV dyssynchrony, that CRT exerts an early and sustained reduction in NT-pro BNP reflecting the improvements in LV geometry and function. Moreover, the Care-HF post-hoc analysis concluded that NT-pro BNP may be used to monitor CRT effect. Consistently, Kubanek et al[18] found similar results and reported that a decrease in BNP > 6.7% between baseline and 3 months follow-up is accurate to differentiate responders from non-responders patients (specificity = 77% and sensibility = 90%). Importantly and in accordance with other studies, our results suggest that baseline neurohumoral activities do not predicts the effect of CRT. Indeed, response to CRT [25] is clearly a multifactor process including the severity of intraventricular asynchrony, presence and localisation of LV viability, and lead placement with respect to the latest LV activation site. In contrast, NT-pro BNP release is essentially determined by LV wall stress. Hence, it is not surprising that baseline NT-pro BNP is not accurate to predict clinical or echocardiographic response after CRT.

### Mechanism of changes in NT-pro BNP following CRT

Mechanism underlying CRT benefit in patients with HF is related to reduction of LV dyssynchrony and corresponding stress-strain disparities and inefficient ventricle contraction. Ultimately, CRT significantly improves both LV function and geometry. This effect results in a decrease in mitral valve tethering and in a concomitant increase in mitral closing forces which, in turn, lead to significant improvement of MR severity. Furthermore, because electrical conduction and regional wall thickening are influenced by the extent of myocardial fibrosis, it has been hypothesized that long-term response to CRT could correlate with myocardial viability in patients with LV dysfunction.

Using nuclear myocardial perfusion imaging (2C1Ti), magnetic resonance imaging, and DSE, several studies have demonstrated the importance of LV viability in predicting response to CRT [26-32]. In accordance with these studies, our results suggest that LV remodeling and decrease in natriuretic peptides post CRT seem to be, at least in part, determined by the presence and extent of myocardial viability. In summary, NT-pro BNP can rapidly drop when cardiac therapy provides significant improvement in LV geometry and function. Obviously, the impact of CRT on both LV and MR is the basic mechanism involved in the change in NT-pro BNP.

### Contractile reserve and response to CRT

Several recent studies had underlined the crucial role of myocardial CR to allow LV reverse remodeling[15,16,29] and clinical improvement[17] following CRT. Moonen et al. [15] showed that response to CRT mainly depends not only on the extent of LV dyssynchrony and MR severity but also on the presence of CR. In addition, CR may be useful to predict CRT response, evaluated either with DSE [33] or with exercise echocardiography[15,16]. Our results confirm that CR is essential to reach satisfactory LV reverse remodeling and response to CRT and that NT pro BNP is an accurate surrogate marker of LV improvement following CRT.

### Clinical implications

BNP is widely considered as useful marker for the diagnosis and prognosis of HF and may be helpful to guide medical management. The presence of high plasma BNP levels is associated with an increased risk of cardiac events and death in patients with chronic HF. The titration of HF treatment based on the reduction of plasma NT-pro BNP concentrations has been found to be superior to treatment with empirical trial-based therapy dictated by clinical judgment. NT-pro BNP is routinely used in patients with HF and is an accurate marker of LV and clinical status.

Recently, Miller et al. [34] found in a prospective series of 172 ambulatory HF patients that a significant increase in BNP was associated with markedly reduced event-free survival. However, only large decrease of BNP concentrations led to outcome improvement. More modest increases or decreases seem to confer little additional predictive value.

Furthermore, our results showed that NT-pro BNP is also a good surrogate marker to identify LV remodeling and MR reduction following CRT. More importantly, NT-pro BNP monitoring allows the clinician to accurately identify echocardiographic responders to CRT. Since LV remodeling predicts outcome with better accuracy than clinical improvement after CRT [35] and that NT-pro BNP monitoring can identify echocardiographic responders with a very good sensitivity and specificity, assessment of NT pro-BNP after CRT could be used as an additional tool to assist the clinician in the evaluation of the patient's condition (additional file 1 and 2). The main finding of this study is that LV remodeling can be identified by change in neurohormone level early (3–6 months) after CRT. Patients identified as non responders may be followed more closely. This subgroup of patients may have a different clinical management: re-evaluation of coronary-sinus lead position, programming of timing intervals, shorter time periods between follow-up evaluations, cardiac transplantation or other surgical options.

### Limitation

The main limitation of this study is the relatively the small number of patients, which reduced the statistical power for multivariate analyses. In addition, the studied population had various LV dysfunction etiologies, including ischemic and non-ischemic myocardial dysfunction. Nevertheless, this also underlines that NT-pro BNP is reliable even in heterogeneous population of CRT patients. Dyssynchrony was defined by longitudinal TDI using a cut-off value of 50 ms on as inclusion criterion. Combining longitudinal and radial dyssynchrony index as inclusion criterion could have been helpful in choosing a more homogenous population prone to CRT response.

## Conclusion

Results of this study show that NT-pro BNP monitoring can accurately identify echocardiographic responders after CRT. Decrease in NT-pro BNP ≥ 15% during follow-up is a good surrogate marker of favourable LV remodeling and MR reduction following CRT. Monitoring of NT-pro BNP may be useful for the management of patients with HF and CRT.

## Competing interests

The authors declare that they have no competing interests.

## Authors' contributions

MS is the principal investigator, he conceived the study and participated in its design and coordination, he participated in the interpretation of the results and he drafts the manuscript; JM performed the statistical analysis, participated in the interpretation of the results and drafts the manuscript; MD participated in data collection, coordination of the study and helped to draft the manuscript; JC participated in the implantation of CRT and in the interpretation of pulmonary X rays for localization of CRT lead; JGD participated to interpreted the results and in the preparation of the manuscript, PP participate in the preparation of the manuscript; FP participated in the implantation of CRT; GO participated in the implantation of CRT; All authors read and approved the final manuscript.

## Supplementary Material

Additional file 1 **Movie representing an echocardiography pre CRT**. This echocardiography is showing a severe mitral regurgitation (i.e. ERO = 42 mm^2^) in a patient with pre CRT BNP level of 1543 pg.ml^-1^. Click here for file

Additional file 2 **Movie representing an echocardiography post CRT**. This echocardiography is showing a mild mitral regurgitation (i.e. ERO = 12 mm^2^) in a patient with post CRT BNP level of 1104 pg.ml^-1 ^(decrease of 27%). Click here for file
